# Fungal Endophyte Colonization Patterns Alter Over Time in the Novel Association Between *Lolium perenne* and *Epichloë* Endophyte AR37

**DOI:** 10.3389/fpls.2020.570026

**Published:** 2020-10-29

**Authors:** Flavia Pilar Forte, Jan Schmid, Paul P. Dijkwel, Istvan Nagy, David E. Hume, Richard D. Johnson, Wayne R. Simpson, Shaun M. Monk, Ningxin Zhang, Tina Sehrish, Torben Asp

**Affiliations:** ^1^Center for Quantitative Genetics and Genomics, Aarhus University, Slagelse, Denmark; ^2^School of Fundamental Sciences, Massey University, Palmerston North, New Zealand; ^3^Ferguson Street Laboratories, Palmerston North, New Zealand; ^4^AgResearch, Grasslands Research Centre, Palmerston North, New Zealand; ^5^Grasslanz Technology, Lincoln, New Zealand

**Keywords:** *Lolium perenne*, *Epichloë endophyte* AR37, coadaptation, seed maintenance program, artificial association, fungal colonization

## Abstract

Infection of the pasture grass *Lolium perenne* with the seed-transmitted fungal endophyte *Epichloë festucae* enhances its resilience to biotic and abiotic stress. Agricultural benefits of endophyte infection can be increased by generating novel symbiotic associations through inoculating *L. perenne* with selected *Epichloë* strains. Natural symbioses have coevolved over long periods. Thus, artificial symbioses will probably not have static properties, but symbionts will coadapt over time improving the fitness of the association. Here we report for the first time on temporal changes in a novel association of *Epichloë* strain AR37 and the *L. perenne* cultivar Grasslands Samson. Over nine generations, a seed maintenance program had increased the endophyte seed transmission rates to > 95% (from an initial 76%). We observed an approximately fivefold decline in endophyte biomass concentration in vegetative tissues over time (between generations 2 and 9). This indicates strong selection pressure toward reducing endophyte-related fitness costs by reducing endophyte biomass, without compromising the frequency of endophyte transmission to seed. We observed no obvious changes in tillering and only minor transcriptomic changes in infected plants over time. Functional analysis of 40 plant genes, showing continuously decreasing expression over time, suggests that adaptation of host metabolism and defense mechanisms are important for increasing the fitness of this association, and possibly fitness of such symbioses in general. Our results indicate that fitness of novel associations is likely to improve over time and that monitoring changes in novel associations can assist in identifying key features of endophyte-mediated enhancement of host fitness.

## Introduction

Seed-transmitted *Epichloë* fungal endophytes, colonizing the intercellular spaces of *Pooideae* grasses, are crucial in determining performance and persistence of pastures in many parts of the world, by protecting their hosts from a range of biotic and abiotic stressors (Clay and Schardl, 2002; Schardl et al., 2004; Sabzalian and Mirlohi, 2010; Xu et al., 2017). However, up until recently, this protection often came at a price to the pastoral industry, in that many of these endophytes produce alkaloids toxic to livestock (Fletcher and Harvey, 1981; Gallagher et al., 1981; Tor-Agbidye et al., 2001; Philippe, 2016). Novel non-toxic *Epichloë*-pasture grass associations have now been established to minimize these detrimental effects (Bouton et al., 2002; Johnson et al., 2013a; Young et al., 2013).

One of the most successful novel associations has been generated by introducing the European *Epichloë* strain AR37 (recently designated to be a member of LpTG-3 (Hettiarachchige et al., 2015) into the New Zealand bred forage *Lolium perenne* cultivar Grasslands Samson (Pennell et al., 2005; Popay and Bonos, 2005; Stewart, 2006; Johnson et al., 2013a). AR37 does not produce the indole diterpene lolitrem B and ergot alkaloids that cause endophyte-associated livestock toxicosis in New Zealand pastures. Instead, it produces several epoxy-janthitrem indole-diterpenes (Tapper and Lane, 2004; Schardl et al., 2012; Panaccione et al., 2014). These, while structurally similar to lolitrem B, are much less harmful to livestock (Thom et al., 2007; Fletcher and Sutherland, 2009; Finch et al., 2013; Thom and Hume, 2013). AR37-grass associations are resistant to five of the six main insect pests of perennial ryegrass (*L. perenne*) in New Zealand, such as African black beetle (*Heteronychus arator*) and root aphid (*Aploneura lentisci*) (Popay and Wyatt, 1995; Hume et al., 2004; Popay and Gerard, 2007; Popay and Thom, 2009); other endophyte metabolites may act in addition to epoxy-janthitrems to confer pest resistance to these associations (Johnson et al., 2013a). Samson was one of the first cultivars sold with AR37, and it has been an important cultivar sold over the last 15 years with this endophyte (Stewart, personal communication).

One of the prerequisites of pastoral use of a novel *Epichloë*/pasture grass association is the continuing annual large-scale production of endophyte-infected seed (Hume et al., 2013; Johnson and Caradus, 2019). A nine-year seed maintenance program has increased AR37-endophyte seed transmission rates in Samson from 76% to close to 100% (see below). This change indicates that some form of coadaptation of the symbionts has occurred, leading to an agronomically desirable outcome—high seed infection rates being crucial to the distribution of the cultivar to farmers (Johnson et al., 2013a; Johnson and Caradus, 2019). However, it seems unlikely that increased seed transmission is the only trait of the association subject to change. Given that interactions between natural associations of seed-transmitted *Epichloë* and their hosts have co-evolved over long periods of time (Schardl et al., 2008; Glémin and Bataillon, 2009), and given the difficulty of establishing novel associations, especially novel associations that meet the requirements for commercial seed production (Christensen et al., 1997; Gagic et al., 2018), it can be assumed that both partners in the AR37/Samson symbiosis were initially not optimally adapted to each other. Since the endophyte depends on the grass for transmission (Saikkonen et al., 2010; Gagic et al., 2018), and since the grass depends on the endophyte for fitness enhancements, it can be assumed that there will be ongoing selection for changes in both symbionts that increase the fitness of the association (Schardl et al., 2004). Such fitness increases may be largely beneficial from an endophyte perspective through increasing pasture productivity, although some, such as increased animal feeding avoidance (Hernández-Agramonte et al., 2018), may not be.

Determining the extent and nature of changes in the AR37/Samson association is thus of interest to breeders and farmers alike, as these changes can be expected to alter the performance of the association. It is also of interest in the context of determining the mechanisms underlying successful interaction between *Epichloë* endophytes and their hosts. Transcriptomic analyses have to date failed to produce a uniform view of these interactions (Dupont et al., 2015; Dinkins et al., 2016; Schmid et al., 2017), and it can be concluded that the symbiosis is finely balanced, with disruption of a large number of endophyte genes associated with catastrophic effects on the symbiosis (Zhang et al., 2006; Tanaka et al., 2008, 2012; Eaton et al., 2010, 2015; Charlton et al., 2012; Johnson et al., 2013b; Chujo and Scott, 2014; Becker et al., 2015; Bassett et al., 2016). As such, genes and features that alter as the AR37/Samson symbiosis evolves, could be implicated as determinants of fitness-enhancing drivers of a successful interaction.

We therefore undertook an analysis of three generations of the initial nine-generation AR37/Samson seed maintenance program to identify changes in both symbionts associated with their co-adaptation.

## Materials and Methods

### Biological Material

Seeds of perennial ryegrass plants of the forage cultivar Grasslands Samson in association with *Epichloë* strain AR37 endophyte (Pennell et al., 2005) from the second, sixth, and ninth generation of a seed maintenance program, described in results (henceforth termed G2, G6, and G9), were obtained from the Margot Forde Germplasm Centre (Palmerston North, New Zealand).

#### Seed Germination

For each generation, 135 to 180 seeds were placed into propagation cell trays (60-cell tray: 6 × 10 cell configuration; 45 mm diameter; 50 mm height; Pöppelmann TEKU, Lohne, Germany) and placed on capillary watered benches in a greenhouse. Each cell was filled with circa 70 mL of potting mix (Daltons, Matamata, New Zealand) augmented with 1.5 g/L of dolomite (Daltons, Matamata, New Zealand) and 1.5 g/L of Lebanon-Turf-Woodace 14-6-11.6 short-term fertilizer (Lebanon Seaboard Cooperation, Lebanon PA, United States). Plants were watered via 2 L/h compensating emitters (Netafim, Auckland, New Zealand) every 2 h for 2 min during day light hours and once overnight. After 3 weeks, germination rates were recorded.

#### Plant Growth, Cloning, and Test for Endophyte Presence

After seed germination, the emerging plants were re-potted in 1 L pots (11 × 11 × 12 cm deep; Primehort, Auckland, New Zealand) filled with potting mix (Daltons) augmented with 1.5 g/L of dolomite (Daltons), 2 g/L of Lebanon-Turf-Woodace 18-2.2-8.3 long-term fertilizer and 1.5 g/L of Lebanon-Turf-Woodace 14-6-11.6 short-term fertilizer (Lebanon Seaboard Cooperation). After 3 weeks, three tillers per plant were tested for presence of endophyte, using a tissue-print immunoassay previously described by Simpson et al. (2012).

Both endophyte-infected and uninfected plants were clonally propagated by placing 10–15 tillers into a new pot (1 L volume, filled with Daltons potting mix augmented with dolomite and fertilizer as described above for 1 L pots) every 6 months; endophyte infection status was assessed for three of these tillers per plant. Plants were placed on capillary watered benches and maintained in the greenhouse with automatic watering, via 2 L/h compensating emitters (Netafim), twice a day for a period of 10 min each time, for the duration of the project. The pots were arranged so that plants from different generations and endophyte-infected and uninfected plant were not separated into different areas; pots were rearranged every 30 days.

### Experimental Setup Under Controlled Conditions

For experiments aimed at investigating plants under controlled environmental conditions, 10 endophyte-infected and three uninfected plants were randomly selected for G2 and G6, while for G9, 11 randomly selected endophyte-infected and two uninfected plants were identified. Three single tillers from each of these plants were potted, individually, in 300 mL trapezoidal root trainers (T2754 Flight-Plastic-Ltd; Lower Hutt, New Zealand) containing potting mix (AgResearch Grasslands, Palmerston North, New Zealand) and grown under controlled conditions (15 ± 2°C, 70% RH, 12 h light, 700 μmol m^–2^ s^–1^) in a plant growth chamber (Contherm BIOSYN Series Model 630). Plants from different generations and endophyte-infected and uninfected plants were randomized. Plants were watered to saturation every other day and the root trainers were rearranged inside the cabinet weekly.

### Plant Growth Analysis

Plant growth was assessed, under controlled environmental conditions, by visually counting, every 2 days, the number of tillers emerging (including leaf tips of new tillers emerging from their mother tiller) from three initial tillers each from 13 G2, 13 G6, and 13 G9 genotypes. For the plant growth curve, the tiller numbers were averaged for each plant genotype and for each time point.

### Microscopic Analysis of Fungal Colonization

In order to determine differences in fungal colonization between generations, one tiller each from 9 G2, 10 G6, and 8 G9 plant genotypes harboring the endophyte, and grown under controlled conditions, was cut at ground level and immediately processed for microscopy analysis.

Pseudostem (the part of the tiller composed of the immature emerging leaf surrounded by the sheaths of the mature leaves) sections of about 1 mm thickness were obtained by making two transverse cuts approximately 1 cm above the tiller base. Sections were initially processed by vacuum fixation with glutaraldehyde (Christensen et al., 2002). Subsequently, sections infiltrated with an acetone/resin mixture were cut into 1-μm slices and stained with 0.05% toluidine blue as previously described (Zhou et al., 2014).

#### Microscopy Data Processing and Statistics

Hyphae in sections were counted at 400× magnification using a Leica light microscope and hyphal diameters were measured using the software ImageJ^1^ (Schneider et al., 2012) in three sections per tiller. For each of the three cross sections, hyphal biovolume was determined by multiplying the averaged area of all the hyphal diameters with the averaged number of hyphae. For all three hyphal measurement, values were averaged for each genotype, and a Kruskal–Wallis test was used to determine statistical significance.

### RNA Extraction

On three occasions, 39 plants per generation, obtained from 13 distinct genotypes per generation, each derived from a different seed, were grown under controlled conditions as described above. From each of the exponentially tillering plants approximately 100 mg (fresh weight) of pseudostem tissue per plant was flash frozen in liquid nitrogen and stored at −80°C. All tillers harvested had two mature leaves. Frozen tissue was ground in liquid nitrogen using a mortar and pestle. RNA extraction was performed using TRIzol reagent (Ambion) following the manufacturer’s protocol. The integrity of the RNA was assessed by 2% agarose gel electrophoresis, while its quantification and purity were measured using a Qubit^®^ 2.0 fluorometer (Thermo Fisher Scientific) and the Qubit^TM^ RNA HS Assay Kit (Life Technologies).

### Transcriptomic Sequencing and Differential Expression Analysis

For each of three biological replicates, equal amounts of RNA from each of the plants of each generation were pooled. Eight, seven, and 11 endophyte-infected plant genotypes were pooled in each of the three biological replicates for G2, G6, and G9, respectively; three endophyte-uninfected plant genotypes for G2 and G6, and two for G9 were pooled for each biological replicate.

Ribonucleic acid sequencing was performed using BGI (Shenzhen, Guangdong, China) by Illumina pair-ended 150 bp sequencing. Raw reads were cleaned from spurious adapter contaminations and low quality terminal nucleotides by Trimmomatic (v0.33, Bolger et al., 2014) and read pairs longer than 60 nt were kept for further processing. A hybrid reference genome consisting of genomic scaffolds of the *E. festucae* strain AR37 genome assembly (Razzaq, 2019) and a *Lolium perenne* genome assembly (Nagy et al. in preparation) was used as the target reference sequence for producing spliced RNA-seq alignments. RNA-seq trimmed reads were mapped to the indexed reference sequences using HISAT2 (Kim et al., 2015). PCR duplicates were removed from the resulting bam files using the bammarkduplicates tool of the biobambam2 package.^2^ Short-read alignments were processed by StringTie (v1.34b, Pertea et al., 2015) to obtain transcript counts and abundances. Gene-based read count information was extracted from the transcript abundance files by the prepDE.py script of the StringTie package. Differential gene expression analysis was carried out by the Bioconductor package EdgeR (Gentleman et al., 2004). From the EdgeR package normalization was applied firstly on the library size by finding a set of scaling factors using a trimmed mean of M-values (TMM) between each pair of samples (Robinson and Oshlack, 2010). Subsequently, the data went through a log2 transformation and normalized by the median of the log2 of the transcript reads per million (RPM). Differential expression of the genes was obtained using a False Discovery Rate (FDR) threshold of 0.05.

## Results

### Endophyte Colonization Changes During Nine Years of Seed Propagation of a Novel Endophyte/Grass Association

**FIGURE 1:**
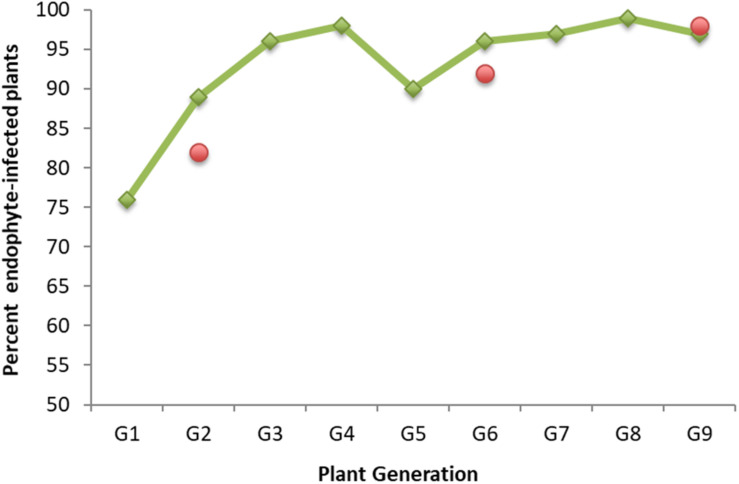
AR37 endophyte seed transmission in perennial ryegrass plants of cultivar Grasslands Samson over a nine-generation seed maintenance program. Shown are the percentages of endophyte-infected plants in each generation (G1–G9) generated from seeds from the previous generation. Presence of the endophyte was assessed by a tissue-print immunoassay (Simpson et al., 2012) or microscopic examination of aniline blue-stained basal leaf sheaths (Simpson et al., 2012). Green rhombs represent infection data generated earlier (Hume et al., unpublished data); red circles data were generated in this study when generating plants from stored seed (see Materials and Methods).

We wanted to explore if and how endophyte colonization changes over time during serial seed propagation of a grass population containing a selected, artificially introduced endophyte. For this purpose, we drew upon stored seed from a nine-generation New Zealand seed maintenance program that improved seed transmission of the endophyte AR37 in the perennial ryegrass cultivar Samson. AR37 had been isolated from a European perennial ryegrass (Popay and Bonos, 2005; Stewart, 2006; Johnson et al., 2013a) and successfully introduced into 38 plants of the cultivar in 1996. Seed transmission increased over a period of nine seed propagation cycles in the maintenance program from 76% in G1 to above 95% in G9 from the offspring of the initial 38 plants (Figure 1). Throughout the program, seed was generated annually by open pollination of plants that had tested positive for endophyte infection, with two exceptions: G3 and G5 were derived from seed generated by open pollination of G2 and G4 plants, respectively, without regard for their endophyte infection status (see Supplementary File 1 for detailed description of the program). While selection of seed for propagation was based entirely on presence of AR37 endophyte (as assessed by immunoassay and strain genotyping), performance of the association in terms of insect resistance has remained unaltered (Popay and Gerard, 2007; Popay and Cox, 2016; Popay personal communication).

Endophyte colonization at three time points in the maintenance program (G2, G6, and G9), was determined for the plants to be used for this work and generated from seed stored under controlled environmental conditions in the Margot Forde Germplasm Centre (Palmerston North, New Zealand). The percentages of plants testing positive for the presence of endophyte were very similar to those determined earlier (Figure 1).

**FIGURE 2:**
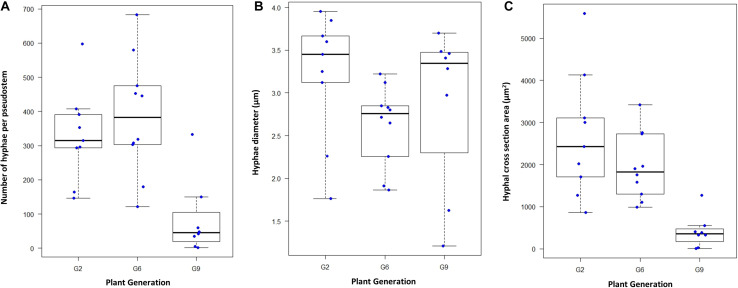
Changes in AR37 colonization over time. **(A)** Box and whisker plot of hyphal numbers determined microscopically in aniline blue-stained 10-micron sheath cross sections (1 mm from the base of the tiller) of the second youngest mature leaf of nine G2 plants, 10 G6 plants and eight G9 plants, grown under controlled conditions. Hyphal numbers used were the averages of three measurements of adjacent sections of the same tiller. **(B)** Average hyphal diameters of hyphae in G2, G6, and G9, respectively, in the same sections (based on measurement of 40–50 hyphae per section). **(C)** Area occupied by hyphae in cross sections, calculated from hyphal numbers and the average hyphal diameters.

**FIGURE 3:**
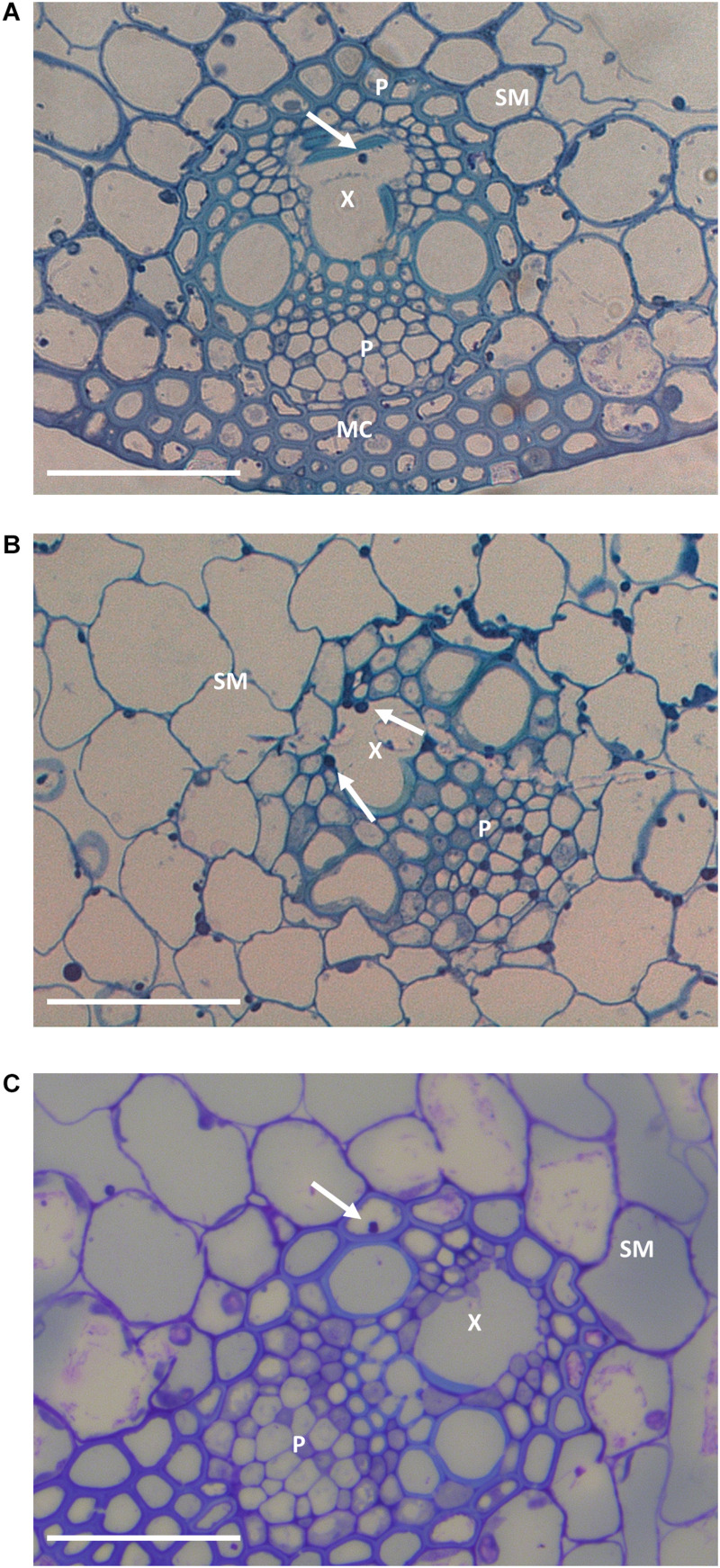
Representative leaf sheath sections in G2 **(A)**, G6 **(B)**, and G9 **(C)** plants. Arrows indicate hyphae within the vascular bundles. In the sections are represented spongy mesophyll (SM), xylem (X), phloem (P), and mesophyll cells (MC). Bar: 50 μm.

In *L. perenne*, *Epichloë* biomass is highest in the leaf sheaths, and levels in any one sheath of a tiller are a reasonable predictor of levels in other parts of the tiller and the overall level of colonization (Herd et al., 1997; Tan et al., 2001; Christensen et al., 2008). Microscopy was used to determine hyphal numbers and hyphal diameters in the sheath of the second youngest mature leaf of exponentially tillering plants grown under controlled conditions using 9, 10, and 8 plants (each from a different seed) for the G2, G6, and G9 generations, respectively; Figures 2, 3. These measurements were also used to assess changes in hyphal biovolume (Figure 2C). All three parameters changed significantly over time (Kruskal–Wallis test with *P* < 0.01, *P* < 0.05, and *P* < 0.01 for numbers, diameter, and combined cross section area, respectively). In particular, hyphal numbers dropped by as much as 75% from G2 to G9, with some G9 plants having < 10 hyphae in the leaf, whereas the minimum number in G2 and G6 was 121 hyphae. Average hyphal diameters also decreased, and as a result of a decline of both number of hyphae and diameter, the average biovolume of the endophyte in the leaf declined by 33% from G2 to G6 and by 81% from G2 to G9.

The frequency of hyphae colonizing vascular bundles is typically increased in incompatible associations (Christensen et al., 1997, 2001). While it remained low overall, it did increase from 0.04% in G2 to 0.23% in G6 and 0.30% in G9 (frequencies were too low to allow us to demonstrate statistical significance at the *P* < 0.05 level; *P* < 0.12; chi-square test).

### The Endophyte’s Transcriptome Did Not Change Across Generations

To investigate whether the transcriptome of the endophyte would reveal adaptation-associated changes in gene expression of the endophyte over time, RNA-seq was performed on pseudostem samples from sets of multiple genetically distinct endophyte-infected plants from each of the three generations. The high-throughput RNA sequencing generated ∼1 GB reads per sample. The total number of 100 bp clean reads produced, after adaptor trimming, ranged from 22,631,866 to 23,849,822 per sample with a number of nucleotide with quality higher than 20/nucleotide (Q20) ranging from 95.93% to 96.56%. The alignment rate was ranging from 78.08% to 80.76%. Trimmomatic was additionally used to remove spurious adapter contaminations and low quality terminal paired-end reads (Bolger et al., 2014). Subsequently, reads with less than 60 nucleotides and unpaired reads were removed. Averaged 16,270,827 RPM, 16,044,654 RPM, and 16,500,651 RPM were mapped on the plant’s genome in G2, G6, and G9, respectively, while averaged 49,404 RPM, 39,817 RPM, and 42,434 RPM were mapped on the endophyte’s genome in G2, G6, and G9, respectively.

A continuous increase or decrease of expression of a gene over time in the maintenance program could be an indication of its role in adaptation. We therefore investigated whether the number of genes for which the number of RPM significantly increased or decreased from G2 to G6 to G9 was higher than expected by chance (Schmid et al., 2017; how this was assessed is described in more detail below for plant genes). This was not the case, which was not unexpected given that the AR37-endophyte propagates clonally (Philipson and Christey, 1986) and different clones are likely to follow different adaptive paths, making clone-specific expression differences difficult to detect in pooled samples.

Expression of janthitrem pathway genes did also not alter significantly as the breeding program progressed (Supplementary Figure 1). Interestingly, the contribution of the endophyte to the transcriptome did also not diminish in spite of the reduction in fungal biomass (Supplementary Figure 2), indicative of an increase of metabolic rate as hyphal number declined, and thus an undiminished capacity of the symbiosis to synthesize janthitrem (Schmid et al., 2017).

### Changes in Plant Response to the Endophyte Over Time

To search for possible signs of host adaptation, growth and plant transcriptomes were monitored over the three generations. There was no detectable change over time for rates of tillering. The length of time before a tiller formed its first daughter tiller was likewise unaltered (Supplementary Figure 3).

Transcriptomic analysis, as previously described by Schmid et al. (2017) identified a small number of host genes whose expression level decreased across the three generations, indicative of adaptation during the maintenance program. Fifty-three genes (binominal confidence interval 40–70) were identified, which had lower RPM values in all three G9 replicates than in all three G6 replicates, with the latter RPM values being also smaller than the RPM values in all three G2 replicates. This number exceeded by 37 the number of genes expected to show a consistent increase or decrease of RPM over time by chance (*z* test; *P* < 0.001; no genes showed consistent increases in RPM over time). To determine which of the 53 genes were most likely to represent the 37 genes that showed expression trends not merely by chance, genes that had a statistically significant expression difference between G2 and G9 were identified using EdgeR. Expression of 40 of the 53 genes met the EdgeR criteria for significant expression differences, matching closely the number of genes (37) predicted to alter expression as a result of adaptation.

For most of the 40 genes, expression changes were subtle. Only for 10 of the genes, expression between the two generations changed more than twofold, but all of these were minor components of the transcriptome (2-27 RPM; Supplementary Tables 1, 2).

**FIGURE 4:**
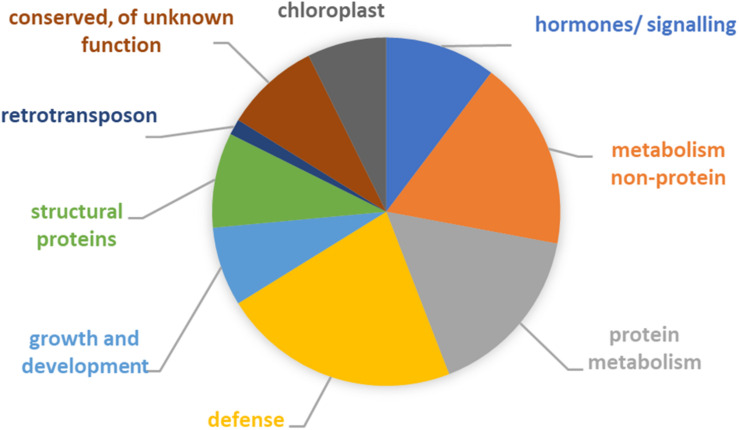
Functional categorization of 40 genes showing an expression signature suggestive of adaptation. See Supplementary Table 1 for details. Note that for some of the genes likely functions involved two or more categories. For six genes, no function could be inferred but all encoded proteins had highly similar homologs in other plant species.

To elucidate which pathways may have altered in the course of the maintenance program, annotation of the 40 differentially expressed genes was performed. The inferred likely functions of these genes (Figure 4 and Supplementary Tables 1, 2) suggested that adaptation of the host involved predominantly changes in metabolism (mostly protein metabolism), as well as hormone metabolism and signaling. Five of the genes had proposed roles in plant cell wall structure, composition, and modification, e.g., extension. Two of the genes, cytochrome P450 and aldo-keto reductase, had likely primary roles in broad defense against biotic and abiotic stresses and plant–microbe interactions (Mierziak et al., 2014; Sengupta et al., 2015; Xu et al., 2015; Tao et al., 2017). In addition, half of the remaining genes had functions that can contribute to plant defense and environmental stress responses (Espartero et al., 1994; Viitanen et al., 1995; Olczak et al., 2003; Afzal et al., 2007; Goff and Ramonell, 2007; Tsai et al., 2012; Antonyuk et al., 2014; Charpentier et al., 2014; Ezaki et al., 2016; Mellidou et al., 2016; Skubacz et al., 2016; Bielach et al., 2017), including chloroplast-associated genes.

## Discussion

New artificial associations between perennial ryegrass and fungal endophytes are being continuously developed (Johnson et al., 2013a) in order to optimize benefits of endophytes to the pastoral industry and to ameliorate negative impacts of climate change. Novel associations are initially intensively tested prior to their commercial release (Thom et al., 2012). Subsequent testing focuses largely on seed transmission of the endophyte, a prerequisite for the bulk generation of endophyte-infected seed (Gagic et al., 2018). It is not known how and to what degree the properties of novel associations change over time—these constitute important issues for long-term agricultural use of such associations, since in a novel symbiosis both partners are expected to undergo adaptive changes in response to selection (Dimijian, 2000). This article describes for the first time changes that occur over generations of a novel association combining the *Epichloë* strain AR37 and the *L. perenne* cultivar Samson (Pennell et al., 2005).

The most notable change was a considerable reduction in endophyte biomass in vegetative tissue, while maintaining a high frequency of seed transmission. Environmental conditions, particularly nitrogen fertilizer, do of course affect endophyte biomass concentration (Stewart, 1986). However, while we did not measure endophyte biomass levels in vegetative tissues under the multitude of field conditions that existed when seed was generated, the decline over time we saw, under controlled conditions, in the three generations, suggests (i) a high adaptive value of minimizing endophyte levels and thus the metabolic burden incurred by its presence and (ii) that a considerable reduction of AR37 endophyte biomass is achievable without compromising seed transmission of the endophyte—and presumably also without compromising protection of the plants from insect pests under field conditions (Popay and Gerard, 2007; Popay and Cox, 2016; Popay personal communication). Our data suggest that the latter may be achieved by increased transcriptomic and metabolic activity of hyphae in the later stages of the breeding program.

A reduction in endophyte biomass did not dramatically alter plant growth, with tillering rates showing no differences between generations. This is not unsurprising as the effects of endophyte colonization on vegetative growth are subtle and complex (Spiering et al., 2006; Tanaka et al., 2006; Eaton et al., 2010). The reduction in biomass could also be considered a sign of increased compatibility: when in reverse genetics experiments the disruption of *Epichloë* genes reduces compatibility with the host, this usually leads to an increase in endophyte biomass (Tanaka et al., 2008; Johnson et al., 2013b; Becker et al., 2015).

Under controlled conditions, as used in our study, it can be demonstrated that the plant genotype (Spiering et al., 2005) and the endophyte strain (Zhang et al., 2006) each play a role in determining endophyte biomass concentration in host tissue. Possibly due to clonal interference, the presence of multiple asexually reproducing lines carrying different mutations (De Visser and Rozen, 2005; Neher, 2013; Vazquez-Garcia et al., 2017), we could not identify any changes in the fungal transcriptome that may have played a role in reducing fungal biomass—there is evidence indicating that AR37 underwent some degree of mutational change during the seed maintenance program, but the number and precise nature of these mutations cannot be inferred with confidence, being bioinformatically derived from pooled clones (Razzaq, 2019). Transcriptome changes in the plant, related to defense mechanisms, suggest that alterations in plant defense mechanisms could play a role in reducing fungal biomass, and this is consistent with the idea that limiting and/or killing of hyphae by the plant could be part of controlling endophyte biomass (Schmid et al., 2017). Other changes may reflect adjustments of the host to AR37 endophyte in photosynthesis (Spiering et al., 2006; Schmid et al., 2017)–chloroplasts act as modulators of plant innate immunity (Padmanabhan and Dinesh-Kumar, 2010)–, hormone production and signal transduction (Dinkins et al., 2016; Schmid et al., 2017), cell wall structure (Dupont et al., 2015), and protein and ion metabolism; some of these are possible secondary effects of reducing endophyte biomass concentration.

In summary, our data support the idea that novel associations continue to evolve over time, presumably toward increased fitness of the symbiosis. Such fitness increases may have an associated agricultural benefit under field conditions, but there is also the possibility of negative effects such as grazing resistance. It is too early to gauge the importance of the effects we saw, in particular the reduction in endophyte biomass under our controlled laboratory conditions, in terms of agronomic performance of pastures. What is clear is that monitoring changes in novel associations may help maintain and optimize their pastoral benefits. In addition, changes in these associations offer a unique opportunity to explore many of the largely unknown mechanisms by which fungal endophytes enhance fitness and pastoral performance of their grass hosts.

## Data Availability Statement

The data for this study have been deposited in the European Nucleotide Archive (ENA) at EMBL-EBI under accession number PRJEB39841 (https://www.ebi.ac.uk/ena/browser/view/PRJEB39841).

## Author Contributions

This study was conceived by TA and JS and coordinated by TA, JS, PD, RJ, and IN. FF, JS, and PD designed the experiments. Endophyte-infected plant material was provided by DH, RJ, WS, and SM. FF collected the data and performed the lab work with contributions from TS and NZ. FF, JS, and IN analyzed the data. The manuscript was written by FF and JS with contributions from DH, RJ, WS, PD, IN, TS, and TA. All authors read the manuscript, contributed to editing, and gave the final approval for publication.

## Conflict of Interest

DH, RJ, and WS are employed by AgResearch Grasslands Research Centre. SM was employed by Grasslanz Technology and currently by NZ WildThings. The remaining authors declare that the research was conducted in the absence of any commercial or financial relationships that could be construed as a potential conflict of interest.

## Footnotes

1 https://imagej.nih.gov/ij/

2 https://gitlab.com/german.tischler/biobambam2

## Supplementary Material

Supplementary File 1 Details of the nine-generation AR37/Samson seed propagation program. AR37, isolated from a European perennial ryegrass, was introduced into 38 *Lolium perenne* plants of the cultivar Grasslands Samson at AgResearch in 1996. These original 38 G0 plants were used to generate new plants (G1) in 1997. The percentage of endophyte-infected plants in G1 was 76% (confidence interval ± 0.14%). In 1999, the G2 generation was produced from the seed of 1200 G1 plants that had been open pollinated at Lincoln. The percentage of endophyte-infected plants in G2 was 89%. Following agronomic trialing in New Zealand, reported elsewhere (Hume et al., 2004, Hume et al., 2007), the G3 was produced in 2007 from the seed of G2 plants following their open pollination at Lincoln using both endophyte-infected and endophyte-free plants. G3 plants were 96% AR37-infected and, following open pollination at Lincoln, seeds from endophyte-infected plants were used to generate G4 (98% endophyte-infected) in 2008. From 2012 to 2016, the population was seed-propagated annually (G5–G9), again using open pollination, at Lincoln. Each year, starting from 2012, only seed from endophyte-infected plants was used to generate the subsequent generation. The percentage of endophyte-infected plants ranged from 90% (G5) to 97% (G9).
