# Supplementary figures and images for: Fungal Endophyte Colonization Patterns Alter Over Time in the Novel Association Between Lolium perenne and Epichloë Endophyte AR37
Source: Front Plant Sci. 2020 Oct 29;11:570026. doi: 10.3389/fpls.2020.570026 (PMC7658011; doi:10.3389/fpls.2020.570026)

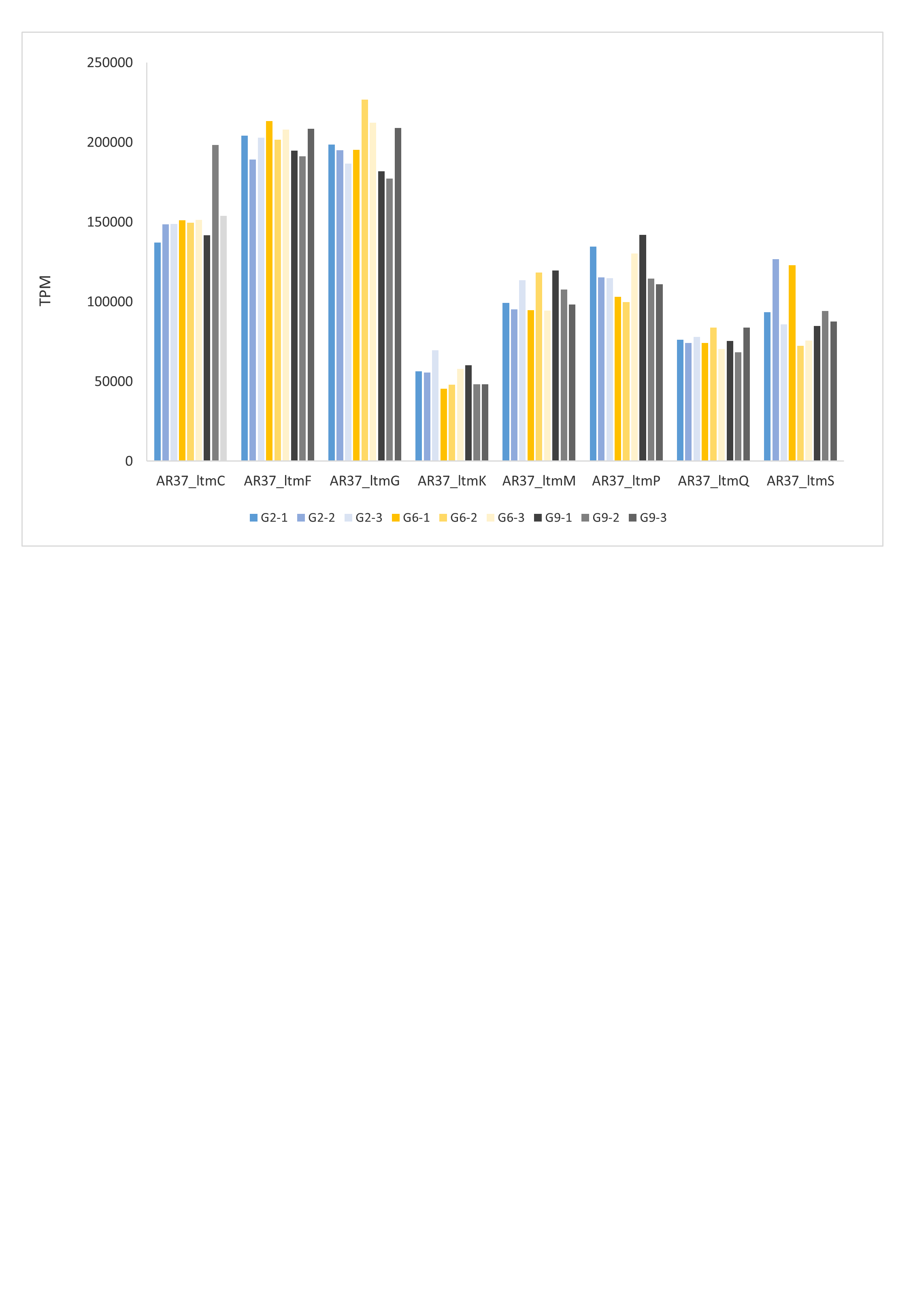

Supplement: Supplementary file 1 [file Image_1.tif]

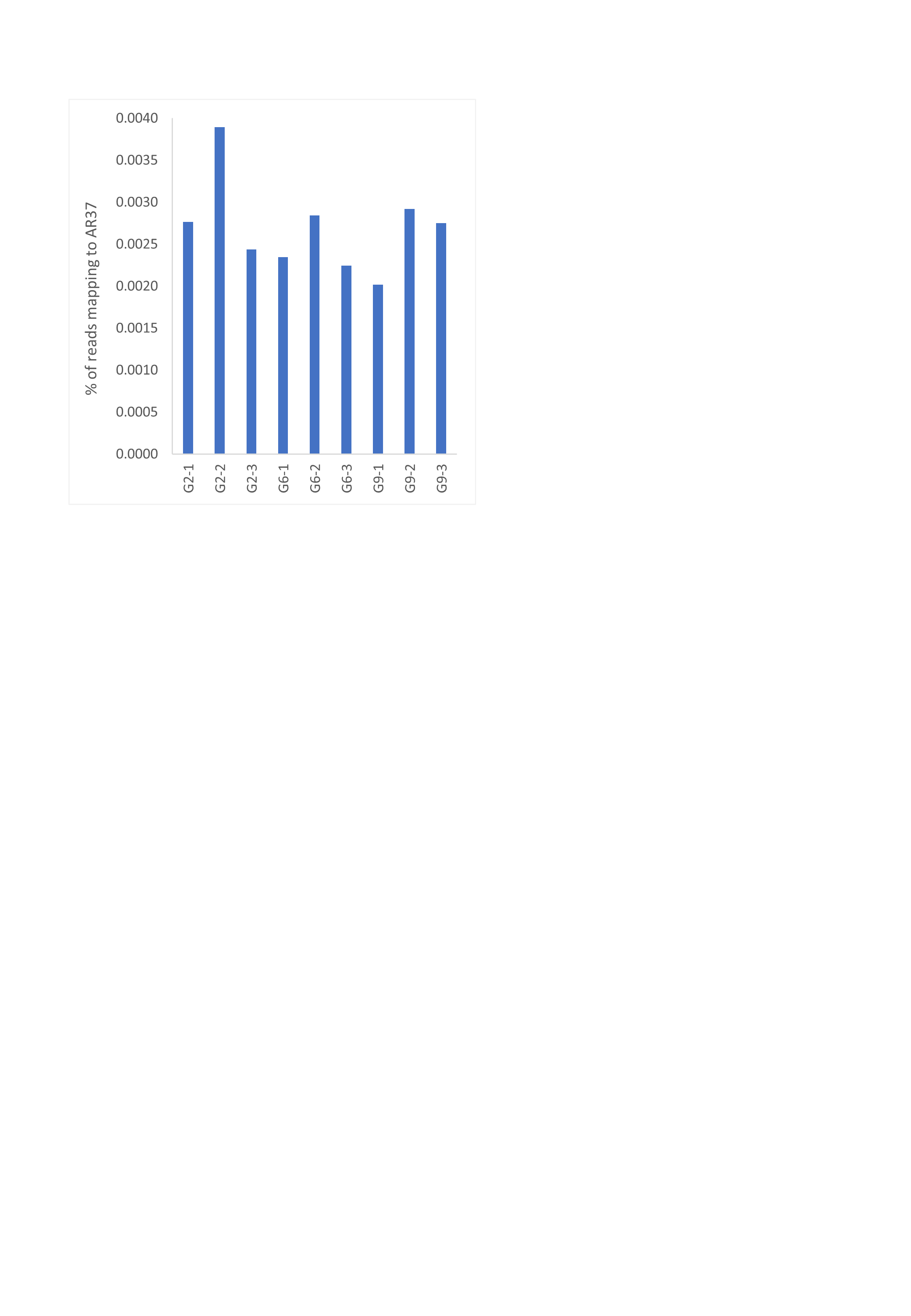

Supplement: Supplementary file 2 [file Image_2.tif]

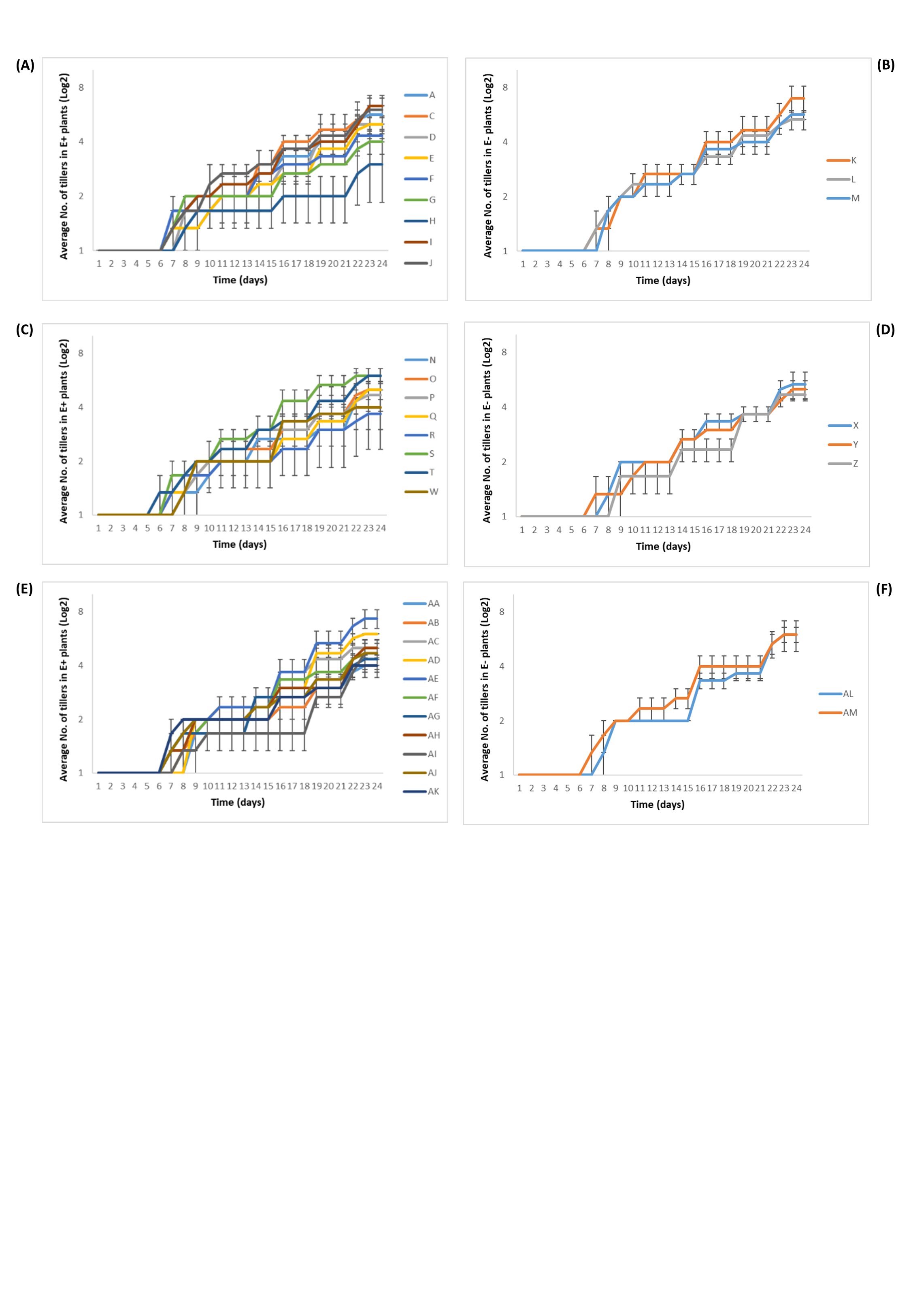

Supplement: Supplementary file 3 [file Image_3.tif]
