# Supplementary material for: Fungal Endophyte Colonization Patterns Alter Over Time in the Novel Association Between Lolium perenne and Epichloë Endophyte AR37
Source: Front Plant Sci. 2020 Oct 29;11:570026. doi: 10.3389/fpls.2020.570026 (PMC7658011; doi:10.3389/fpls.2020.570026)
Supplement: Supplementary File 1 — Details of the nine-generation AR37/Samson seed propagation program. AR37, isolated from a European perennial ryegrass, was introduced into 38 Lolium perenne plants of the cultivar Grasslands Samson at AgResearch in 1996. These original 38 G0 plants were used to generate new plants (G1) in 1997. The percentage of endophyte-infected plants in G1 was 76% (confidence interval ± 0.14%). In 1999, the G2 generation was produced from the seed of 1200 G1 plants that had been open pollinated at Lincoln. The percentage of endophyte-infected plants in G2 was 89%. Following agronomic trialing in New Zealand, reported elsewhere (Hume et al., 2004, Hume et al., 2007), the G3 was produced in 2007 from the seed of G2 plants following their open pollination at Lincoln using both endophyte-infected and endophyte-free plants. G3 plants were 96% AR37-infected and, following open pollination at Lincoln, seeds from endophyte-infected plants were used to generate G4 (98% endophyte-infected) in 2008. From 2012 to 2016, the population was seed-propagated annually (G5–G9), again using open pollination, at Lincoln. Each year, starting from 2012, only seed from endophyte-infected plants was used to generate the subsequent generation. The percentage of endophyte-infected plants ranged from 90% (G5) to 97% (G9). [file Data_Sheet_1.docx]

Supplementary Material

**
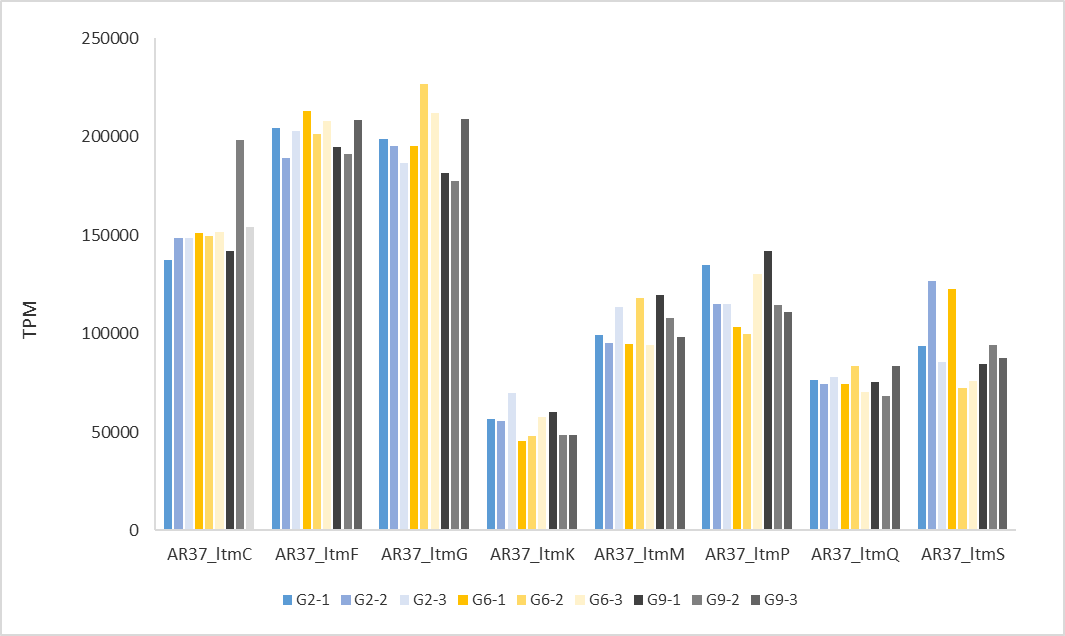
**

**Supplementary Figure 1.** Expression of eight AR37 janthitrem biosynthesis genes (in TPM based on reads mapping to the endophyte genome), identified as homologues lolitrem pathway genes (Razzaq, 2019; Johnson, personal communication). Shown are expression levels for each of three biological replicates in each generation.

**
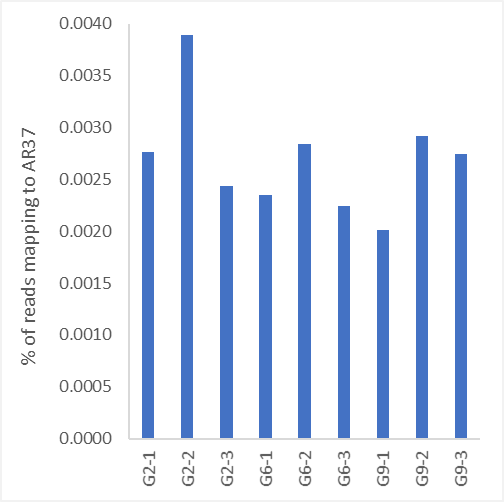
**

**Supplementary Figure 2.** Percentage of all reads mapping to AR37 in each biological replicate of three generations of the seed maintenance program. Labels refer to the Generation, followed by the number of the biological replicate.

##
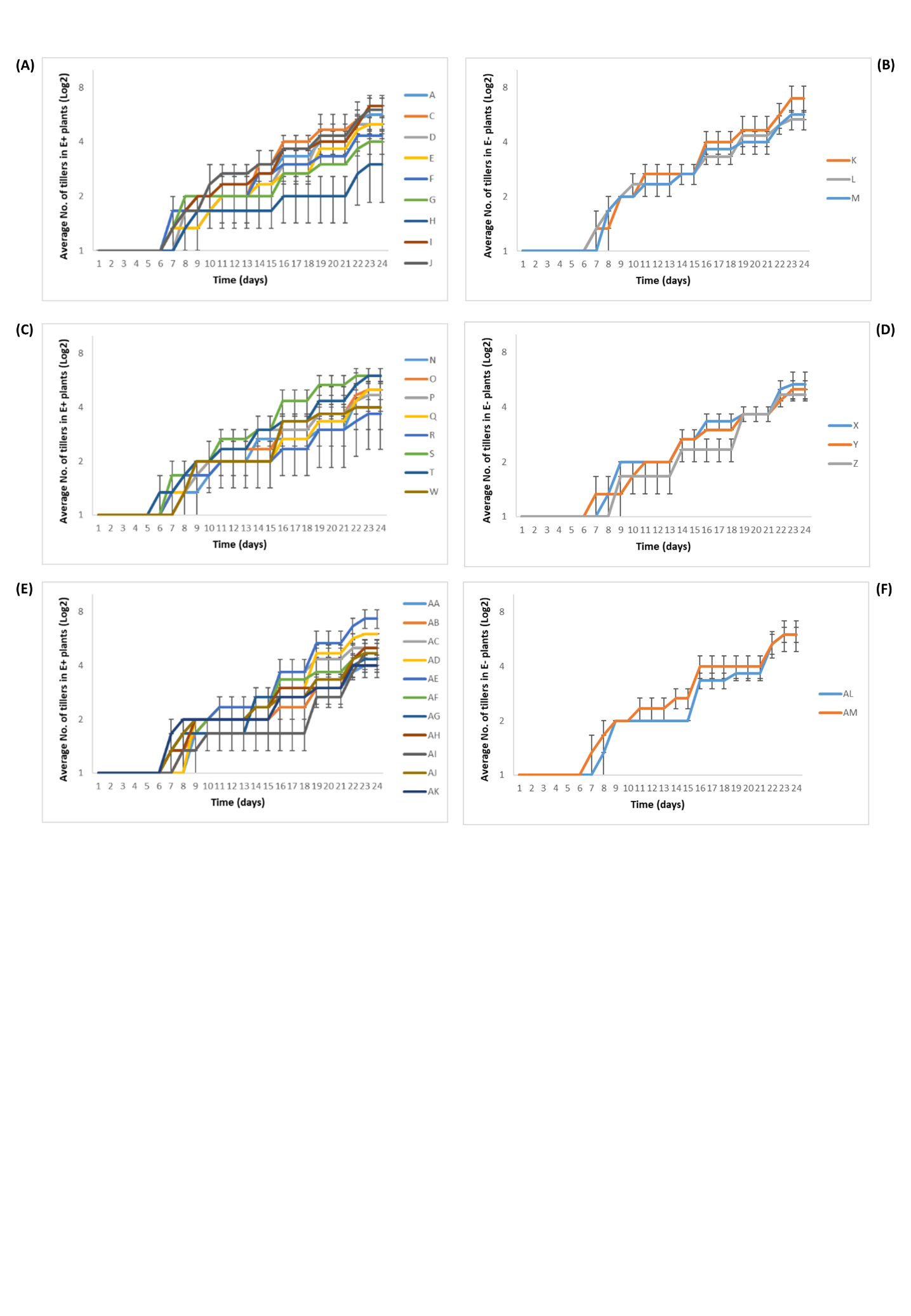


**Supplementary Figure 3.** Plant growth curves in G2 (A, B), G6 (C, D) and G9 (E, F). Single tillers of endophyte-infected and endophyte-free plant genotypes in three biological replicates were placed into root trainers and grown under controlled conditions in a growth cabinet. Plant growth was measured by tiller counting every other day. (A, B) Graphs displaying nine endophyte-infected (E+) (individual plants for one of the genotypes died) and three endophyte-free (E-) plant genotypes for G2. (C, D) Eight E+ (individual plants for two of the genotypes died) and three E- plant genotypes for G6. (E, F) Eleven E+ and two E- genotypes for G9. Weighted average log phases (appearance of the first daughter tiller) were 7 days for both G6 and G9, and for both E+ and E-. For G2, the weighted average log phase was 6 days for both E+ and E-. Error bars represent Standard Errors of the three biological replicates.
